# Exploring the dynamics of vascular adaptation

**DOI:** 10.7554/eLife.88052

**Published:** 2023-05-25

**Authors:** Thomas S Shimizu, E Toby Kiers, Howard A Stone

**Affiliations:** 1 AMOLF Amsterdam Netherlands; 2 Amsterdam Institute for Life and Environment, Vrije Universiteit Amsterdam Amsterdam Netherlands; 3 Department of Mechanical and Aerospace Engineering, Princeton University Princeton United States

**Keywords:** network, P. polycephalum, morphogenesis, vasculature, time delay, adaptation, Other

## Abstract

A combination of *in toto* imaging and theory suggests a new mechanism for the remodeling of veins in vascular networks.

**Related research article** Marbach S, Ziethen N, Bastin L, Bäuerle FK, Alim K. 2023. Vein fate determined by flow-based but time-delayed integration of network architecture. *eLife* **12**:e78100. doi: 10.7554/eLife.78100.

Many processes in biology rely on molecules and other objects moving from A to B. These movements are dominated by diffusion at the smallest scales (Berg, 1993), but diffusion is prohibitively slow for distances greater than a few millimeters, so nature relies on the directed flow of fluids instead. The best-known example of this is the use of blood to transport nutrients, gases and waste materials around the body (Wootton and Ku, 1999). However, vascular networks have evolved many times across diverse domains of life: even microbes such as bacteria (Claessen et al., 2014; Wilking et al., 2013) and fungi (Fricker et al., 2017b; Whiteside et al., 2019) are known to build fluid flow networks for the long-range transport of nutrients and other substances within colonies. Given the importance of vascular networks for so many organisms, a natural question arises: what are the unifying principles, if any, that govern the design of such networks?

One possible answer to this question was proposed by Cecil D Murray of Bryn Mawr College nearly a century ago (Murray, 1926). By considering the energetic costs of pressure-driven blood flow, Murray identified a simple relationship between three physical parameters (the radius of the blood vessel, *a*; the flow rate, *Q*; and the viscosity of blood, *µ*), and a single physiological parameter (the rate of energy dissipation required to maintain a vein, per unit volume, *b*). This relationship – now known as Murray’s law – can be written as: *a*^6^=16*µQ*^2^/π^2^*b*.

Since then, various predictions that follow from Murray’s law have been shown to hold for a range of vascular networks in animals (West et al., 1997), plants (McCulloh et al., 2003) and microbes (such as the slime mold *Physarum polycephalum*; Fricker et al., 2017a). However, Murray’s law only applies to flow networks that do not change with time, and it does not address how such networks are built in the first place, or how they remodel themselves in response to anatomical and/or physiological changes. A lack of high-quality data on vascular flows and morphology at the scale of the entire network has hindered efforts to understand how vascular networks grow and remodel themselves over time.

Now, in eLife, Karen Alim (Technical University of Munich) and co-workers – including Sophie Marbach (New York University) and Noah Ziethen (Max Planck Institute for Dynamics and Self-Organization) as joint first authors, Leonie Bastin and Felix Bäuerle – report the results of extensive experimental and theoretical work to understand how the vascular networks formed by *P. polycephalum* change over time (Marbach et al., 2023b).

Combining high-resolution measurements of flow inside individual veins with time-lapse imaging of entire networks (Figure 1), Marbach et al. first discovered that the radius of a vein changes when the local shear rate of the fluid flow changes, albeit with a time delay. The researchers then plotted the trajectories of individual veins in a “phase space” with vein radius as the x-axis and shear rate as the y-axis. This revealed a rich repertoire of dynamical behaviors, such as oscillating orbits and runaway decays. Moreover, these behaviors could be classified into several categories, depending on the eventual fate of the vein (e.g., some veins were stable and supported contractile oscillations, whereas others were unstable and shrunk to extinction) and other factors. Such rich dynamics cannot be explained by Murray’s law, which predicts only a fixed relationship between the shear rate and vein radius.

**Figure 1.**
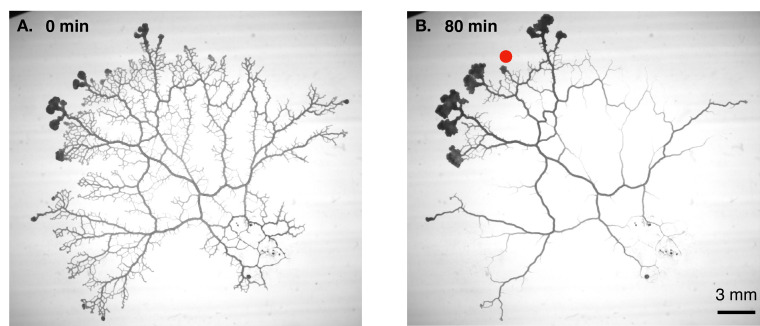
Vascular remodelling observed in real time. (**A**) Image showing the vascular network formed by the slime mould *Physarum polycephalum*. (**B**) Image of the same network 80 minutes later. Note that many veins have disappeared, whereas others have maintained a constant thickness, and some have become thicker. The new vascular adaptation model developed by Marbach et al. can predict these contrasting fates for individual veins in the network. Red dot shows veins where shear rate is initially low.

Marbach et al. then developed a detailed mathematical framework that consisted of three parts: (i) a model of how the vein radius responds to changes in the shear rate that takes into account the observed time delay and force balance in wall of the vein; (ii) a model of fluid flows in contractile veins; (iii) a method to extract certain network parameters that are required to apply the vein adaptation model (i) and the flow model (ii) to experimental data. The method used to extract these parameters – which reflect the global topology and morphology of the network – involved the application of Kirchoff’s circuit laws to fluid flow networks.

A key ingredient of the new model is a mechanism for sensing the local shear rate. This mechanism relies on a peculiar feature of the actin cytoskeletal networks that drive the contractile dynamics of veins in *P. polycephalum* (Isenberg and Wohlfarth-Bottermann, 1976). Specifically, it has been shown that networks of semi-flexible polymers such as actin can contract in response to shear stresses, in a direction that is, counter-intuitively, orthogonal to the direction of the applied shear (Janmey et al., 2007). The researchers argue that this property of actin can provide a mechanism that enables local shear (due to fluid flow along the long axis of a vein) to lead to an increase in the radius of the vein (see the accompanying preprint; Marbach et al., 2023a).

Through extensive mathematical analyses, Marbach et al. succeeded in reducing these detailed considerations to an elegant model consisting of just three equations: one describing the shear-rate feedback (via the actin cytoskeleton), a second describing the observed time delay at a phenomenological level, and a third describing the dependence of local shear rate on the global architecture of the network. Despite its simplicity, when calibrated by flow-resistance calculations based on morphological data for the full network, this model faithfully reproduced the rich set of dynamics observed for individual veins over time, including their eventual fate – that is, whether they are stable and oscillate about a fixed point (with this point being predicted by Murray’s law), or are unstable and shrink to extinction over time.

Crucial for the successful validation and application of the model was the acquisition of high-quality spatial and temporal data on the detailed shape of the *P. polycephalum* network *in toto* – that is, across the entirety of the network. It is challenging to collect data of such completeness for vasculature studies in macroscopic organisms. However, advances in the imaging of large volumes (Stephan et al., 2019) and the advent of organoid models of vasculature (Wimmer et al., 2019) raise the exciting prospect that it may soon become possible to test the generality of this new model in other systems.

Equally compelling are the implications of the latest findings in the broader context of organism-scale behavior in natural environments. Given there are ~900 different species of slime molds of various shapes, structure and sizes (Meena et al., 2022), this new model provides a foundation for asking previously unimagined questions about how vascular adaptation dynamics enables, and is in turn affected by, a dynamic networked anatomy that captures and feeds on microbial prey while navigating through ecosystems.
